# The correlation between TyG-BMI and the risk of osteoporosis in middle-aged and elderly patients with type 2 diabetes mellitus

**DOI:** 10.3389/fnut.2025.1525105

**Published:** 2025-03-11

**Authors:** Yanrong Chen, Yindi Zhang, Si Qin, Fadong Yu, Yinxing Ni, Jian Zhong

**Affiliations:** Department of Endocrinology, The Third Affiliated Hospital of Chongqing Medical University, Chongqing, China

**Keywords:** type 2 diabetes mellitus, osteoporosis, insulin resistance, triglyceride, glucose, body mass index, bone mineral density

## Abstract

**Background and objectives:**

Osteoporosis (OP) has emerged as one of the most rapidly escalating complications associated with diabetes mellitus. However, the potential risk factors contributing to OP in patients with type 2 diabetes mellitus (T2DM) remain controversial. The aim of this study was to explore the relationship between triglyceride glucose-body mass index (TyG-BMI), a marker of insulin resistance calculated as Ln [triglyceride (TG, mg/dL) × fasting plasma glucose (mg/dL)/2] × BMI, and the risk of OP in T2DM patients.

**Methods:**

This retrospective cross-sectional study enrolled 386 inpatients with T2DM, comprising both male and postmenopausal female participants aged 40 years or older. Individuals with significant medical histories or medications known to influence bone mineral density were excluded. Machine learning algorithms were employed to rank factors affecting OP risk. Logistic regression analysis was performed to identify independent influencing factors for OP, while subgroup analysis was conducted to evaluate the impact of TyG-BMI on OP across different subgroups. Restricted cubic spline (RCS) analysis was used to explore the dose-response relationship between TyG-BMI and OP. Additionally, the receiver operating characteristic (ROC) curve was utilized to assess the predictive efficiency of TyG-BMI for OP.

**Results:**

Machine learning analysis identified TyG-BMI as the strongest predictor for type 2 diabetic osteoporosis in middle-aged and elderly patients. After adjusting for confounding factors, multivariate logistic regression analysis revealed that age, osteocalcin, and uric acid were independent influencing factors for OP. Notably, TyG-BMI also emerged as an independent risk factor for OP (95%CI 1.031–1.054, *P* < 0.01). Subgroup analysis demonstrated a consistent increase in OP risk with higher TyG-BMI levels across all subgroups. RCS analysis indicated a threshold effect, with the risk of OP gradually increasing when TyG-BMI exceeded 191.52. Gender-specific analysis showed increasing the risk of OP when TyG-BMI surpassed 186.21 in males and 198.46 in females, with a more pronounced trend observed in females. ROC suggested that TyG-BMI index has significant discriminative power for type 2 diabetic osteoporosis.

**Conclusion:**

TyG-BMI has been identified as a robust predictive biomarker for assessing OP risk in middle-aged and elderly populations with T2DM.

## Introduction

Diabetes mellitus is a global health issue, with an adult prevalence rate of 11.2% in China and a staggering 20% among individuals aged 60 and older (1). Type 2 diabetes mellitus (T2DM) accounts for over 90% of diabetes cases, placing a significant burden on both the economy and healthcare systems. T2DM and its associated metabolic disturbances can disrupt bone metabolism through various mechanisms, potentially leading to skeletal complications such as osteoporosis (OP) (2). Previous studies have shown that T2DM increases the risk of bone damage and fractures (3, 4). In mainland China, the pooled prevalence rate of OP among T2DM patients is more than one-third, with higher risks observed among female and elderly patients (5). Among T2DM patients, bone fractures are not only challenging to treat but also time-consuming, and may even lead to severe complications, resulting in elevated rates of disability and mortality. Therefore, further research could enable the development of a tool or biomarker to predict the risk of OP in T2DM patients, facilitating targeted bone health management strategies.

Insulin resistance (IR), which may disrupt the normal balance of bone formation and resorption and affect calcium (Ca) and phosphorus (P) metabolism, is one of the core pathological mechanisms in the pathogenesis of T2DM. In the early stage of the pathophysiological process of T2DM, characterized by IR and hyperinsulinemia, the bone mineral density (BMD) of T2DM patients is normal or even elevated, but bone strength decreases. This leads to a higher risk of fragility fracture compared to non-T2DM patients (6). Studies have indicated that surrogate marker of IR may have a negative impact on BMD in T2DM patients (7), while it is positively correlated with BMD in postmenopausal patients with T2DM (8). Currently, the mechanism by which IR causes diabetes-related bone damage remains unclear, and there are few studies specifically addressing IR and type 2 diabetic osteoporosis. Therefore, it is particularly important to explore the relationship between IR and OP in T2DM patients.

Although the hyperinsulinemic-euglycemic clamp (HEC) remains the gold standard for IR assessment, its clinical application is limited by technical complexity, high costs, and poor patient compliance. As a result, some simplified and practical IR assessment indices, based on conventional biochemical or anthropometric parameters and requiring no serum insulin measurement, have been gradually developed and are now widely applied in clinical practice. Examples include the triglyceride glucose (TyG) index and the triglyceride glucose-body mass index (TyG-BMI) (9). A retrospective cross-sectional study has reported that IR, as indicated by TyG, is significantly associated with an increased risk of low bone mass and OP (10). TyG-BMI has shown better early diagnostic efficacy for IR screening compared to the single determination of TyG and other combined detection indicators (11). To date, no studies have investigated the correlation between TyG-BMI and diabetic osteoporosis. Therefore, we speculate that TyG-BMI may hold promise as a potential predictor of OP risk in T2DM patients.

In diabetic patients, dual X-ray absorptiometry (DXA) is the most commonly used clinical tool for assessing OP; however, factors such as degenerative changes in the examined areas, calcification in surrounding soft tissues, and BMI may lead to false-negative diagnoses (12). In recent years, quantitative computed tomography (QCT) has been gradually used in clinical practice to avoid measurement errors caused by vascular calcification, vertebral degeneration or body weight, thereby providing a more sensitive assessment of OP. A cross-sectional study involving diabetic patients who underwent both spinal QCT and hip DXA examinations confirmed the diagnostic accuracy of QCT in detecting OP in diabetic patients, indicating that QCT is an excellent diagnostic tool (13).

Therefore, this study employs QCT as the diagnostic criterion for OP and conducts a retrospective analysis to identify the influencing factors of type 2 diabetic osteoporosis in middle-aged and elderly patients. We aim to establish a reliable clinical foundation for bone health management by exploring the association between TyG-BMI and the risk of OP in this population with T2DM.

## Subjects and methods

### Subjects

A total of 386 T2DM participants admitted to the Endocrine Disease Center of the Third Affiliated Hospital of Chongqing Medical University between June 2022 and December 2023 were retrospectively enrolled. All participants received standardized medication treatment for T2DM during their hospitalization. This study, conducted in accordance with the Declaration of Helsinki, was approved by the Institutional Ethics Committee of the Third Affiliated Hospital of Chongqing Medical University for retrospective analysis (Ethics number: 2023-KL-91).

### Inclusion and exclusion criteria

Inclusion criteria: (1) age ≥ 40 years old; (2) diagnosis of T2DM according to the American Diabetes Association (ADA) standards (14): fasting plasma glucose (FPG) ≥ 7.0 mmol/L, 2-h plasma glucose ≥ 11.1 mmol/L during oral glucose tolerance test or random plasma glucose ≥ 11.1 mmol/L, glycosylated hemoglobin (HbA1c) ≥ 6.5%; (3) all female participants were postmenopausal women.

Exclusion criteria: (1) patients with thyroid diseases, parathyroid diseases, rheumatic diseases, malignancies or prolonged immobilization that may affect bone metabolism; (2) use of medications affecting bone metabolism such as estrogens, glucocorticoids, bisphosphonates, calcitonin, thiazolidinediones, immunosuppressants, anticonvulsants, or antidepressants; (3) patients with a history of severe hepatic or renal dysfunction, acute infection, trauma, or surgical interventions within the past 3 months.

### Data collection

General data and laboratory examinations: General data were collected for all enrolled participants, including gender, age, smoking and drinking history, and diabetes duration. All data were meticulously extracted from the hospital’s electronic medical records. Participants underwent height and weight measurements while wearing light indoor clothing and in an empty stomach, with BMI subsequently calculated. Blood pressure in calm state was measured twice, and the average systolic blood pressure (SBP) and diastolic blood pressure (DBP) values were recorded. Fasting venous blood samples were collected the next morning after admission. Laboratory analyses included measurements of FPG, HbA1c, glycated albumin (GA), total cholesterol (TC), triglyceride (TG), high-density lipoprotein cholesterol (HDL-C), low-density lipoprotein cholesterol (LDL-C), procollagen type 1 N-terminal propeptide (P1NP), β-C-terminal telopeptide of type I collagen (β-CTX), osteocalcin (OC), bone-specific alkaline phosphatase (BAP), 25-hydroxyvitamin D (25OHD), parathyroid hormone (PTH), calcitionin, serum Ca, serum P, serum albumin (ALB), serum uric acid (UA), serum creatinine (SCr), and glomerular filtration rate (eGFR).

BMI is calculated by dividing weight (in kilograms) by the square of height (in meters) (kg/m^2^). TyG-BMI is calculated using the formula: Ln [TG (mg/dL) × FPG (mg/dL)/2] × BMI. To ensure the accuracy of data extraction, the data were entered into a spreadsheet by one researcher and cross-checked by another independent researcher.

### Diagnostic criteria

The lumbar BMD was measured using QCT (3001 S Lamar Blvd Ste 302, Austin, TX 78704, QCT PRO V6.1). The diagnostic criteria for QCT osteoporosis of the lumbar spine followed the guidelines of the International Society for Clinical Densitometry (ISCD) (15): The mean values of BMD of the two lumbar cancellous bones (usually the first and second lumbar vertebrae) were calculated, and the absolute BMD from lumbar QCT was used for diagnosis. An absolute BMD value greater than 120 mg/cm^3^ is considered normal bone mass, a value between 80 and 120 mg/cm^3^ is classified as osteopenia, and a value less than 80 mg/cm^3^ indicates OP. Notably, the QCT diagnostic criteria for OP proposed by ISCD have been validated through extensive QCT data analysis in the Chinese population and are deemed applicable to this demographic as well (16, 17).

### Statistical analysis

Counting data were represented as N (%), and the Chi-square test was used for comparisons between groups. Normally distributed continuous data were expressed as mean ± standard deviation (SD), and the independent samples *t*-test was used for comparisons between groups. Non-normally distributed continuous data were expressed as *M (P25, P75)*, and the non-parametric Mann-Whitney U test was used for comparisons between groups. First, based on machine learning algorithms, decision trees, random forest (RF), and extreme gradient boosting (XGBoost) were constructed to rank the influencing factors of OP in middle-aged and elderly T2DM patients. Second, multivariable logistic regression analysis was employed to identify the independent influencing factors of type 2 diabetic osteoporosis, and the influence of TyG-BMI on OP in different subgroups was evaluated by subgroup analysis. Additionally, restricted cubic splines (RCS) was utilized to explore potential nonlinear associations between different levels of TyG-BMI and OP. Finally, the receiver operating characteristic (ROC) curve was used to evaluate the predictive efficacy of TyG-BMI for OP. Statistical analyses were conducted using R software, version 3.6.1, and SPSS 26.0 software (SPSS, IBM Corp., Armonk, NY, USA). GraphPad Prism 8.0 (GraphPad Software, Inc., San Diego, CA) was used for generating the image. A *P*-value < 0.05 was considered statistically significant.

## Results

**FIGURE 1:**
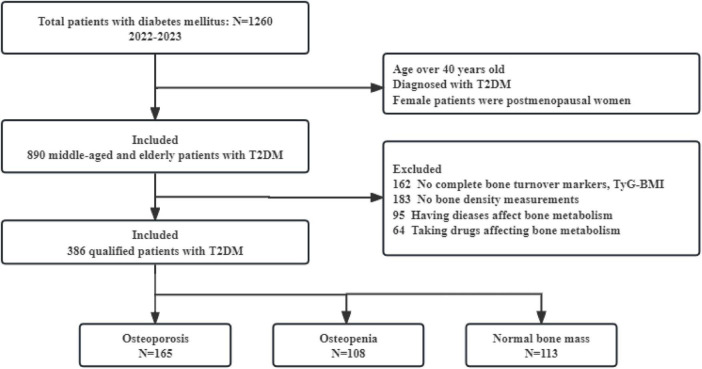
Flow chart of this study. T2DM, type 2 diabetes mellitus; TyG-BMI, triglyceride glucose-body mass index.

The study flowchart was summarized in Figure 1.

### Comparison of baseline characteristics between the OP group and non-OP group

A total of 386 subjects were included in this study, comprising 181 males and 205 females, with a mean age of 62.5 ± 10.2 years, a mean BMI of 24.3 ± 3.8 kg/m^2^, and a mean diabetes duration of 5.0 (1.0, 10.0) years. Based on the aforementioned criteria, BMD measurements revealed that 42.7% (165 cases) had OP, 28.0% (108 cases) had osteopenia, and 29.3% (113 cases) had normal bone mass.

No significant differences were observed between the OP group and the non-OP group in DBP, TC, LDL-C, HDL-C, β-CTX, 25OHD, PTH, calcitonin, Ca, P, ALB, SCr and eGFR. In the OP group, age, diabetes duration, BMI, SBP, FPG, HbA1c, GA, TG, P1NP, OC, BAP and TyG-BMI were significantly higher than those in the non-OP group, while UA, lumbar spine QCT values, smoking and drinking were lower than those in the non-OP group (all *P* < 0.05) (Table 1).

**TABLE 1:** Comparison of general data between the non-OP group and the OP group.

Variable	Non-OP group (*n* = 221)	OP group (*n* = 165)	*P-*value
Gender (*n* %)			**<0.001**
Female	90 (40.72)	115 (69.70)	
Male	131 (59.28)	50 (30.30)	
Age (years)	58.50 ± 9.26	68.00 ± 8.73	**<0.001**
Diabetes duration (years)	5.00 (1.00, 10.00)	7.00 (2.00, 12.00)	**0.03**
Smoking (*n* %)			**0.007**
Yes	142 (64.25)	128 (77.58)	
No	79 (35.75)	37 (22.42)	
Drinking (*n* %)			**<0.001**
Yes	130 (58.82)	126 (76.36)	
No	91 (41.18)	39 (23.64)	
BMI (kg/m^2^)	22.9 ± 3.15	26.3 ± 3.72	**<0.001**
SBP (mmHg)	131 ± 17.3	138 ± 20.0	**0.001**
DBP (mmHg)	80.5 ± 12.3	80.6 ± 10.9	0.916
FPG (mmol/L)	9.13 ± 4.25	11.7 ± 3.86	**<0.001**
HbA1c (%)	9.10 ± 2.27	9.99 ± 2.44	**<0.001**
GA (%)	23.9 ± 8.29	27.7 ± 10.4	**<0.001**
TG (mmol/L)	2.12 ± 1.72	3.05 ± 1.85	**<0.001**
TC (mmol/L)	4.70 ± 1.32	4.91 ± 1.36	0.114
HDL-C (mmol/L)	1.10 ± 0.34	1.16 ± 0.39	0.089
LDL-C (mmol/L)	2.77 ± 0.96	2.98 ± 1.36	0.08
β-CTX (ng/mL)	0.25 (0.16, 0.41)	0.28 (0.18, 0.43)	0.115
P1NP (ng/mL)	40.0 ± 18.5	44.1 ± 20.7	**0.047**
OC (ng/mL)	11.5 ± 4.89	13.5 ± 6.80	**0.001**
BAP (ug/L)	16.0 ± 6.03	17.9 ± 7.60	**0.008**
25OHD (ng/mL)	18.9 ± 7.65	17.9 ± 7.31	0.201
PTH (pg/ml)	43.4 ± 17.7	43.2 ± 21.2	0.92
Calcitionin (pg/ml)	1.66 ± 1.98	1.67 ± 1.80	0.936
Ca (mmol/L)	2.28 ± 0.15	2.28 ± 0.15	0.849
P (mmol/L)	1.13 ± 0.19	1.13 ± 0.19	0.971
UA (umol/L)	337 ± 87.3	304 ± 88.4	**<0.001**
ALB (g/L)	40.8 ± 4.62	40.7 ± 4.16	0.846
SCr (umol/L)	67.4 ± 28.5	67.3 ± 24.6	0.955
eGFR (mL/min/1.73 m^2^)	110 ± 34.9	110 ± 31.9	0.942
QCT (mg/cm^3^)	125 ± 27.8	62.8 ± 14.3	**<0.001**
TyG-BMI	178 ± 33.3	223 ± 38.5	**<0.001**

Boldface indicates statistical significance (*P* < 0.05). OP, osteoporosis; BMI, body mass index; SBP, systolic blood pressure; DBP, diastolic blood pressure; FPG, fasting plasma glucose; HbA1c, glycosylated hemoglobin; GA, glycated albumin; TG, triglyceride; TC, total cholesterol; HDL-C, high-density lipoprotein cholesterol; LDL-C, low-density lipoprotein cholesterol; β-CTX, β-C-terminal telopeptide of type I collagen; P1NP, procollagen type 1 N-terminal propeptide; OC, osteocalcin; BAP, bone-specific alkaline phosphatase; 25OHD, 25-hydroxyvitamin D; PTH, parathyroid hormone; Ca, serum calcium; P, serum phosphorus; UA, serum uric acid; ALB, serum albumin; SCr, serum creatinine; eGFR, glomerular filtration rate; QCT, quantitative computed tomography; TyG-BMI, triglyceride glucose-body mass index.

### Screening and analysis of clinical factors for type 2 diabetic osteoporosis in middle-aged and elderly patients

**FIGURE 2:**
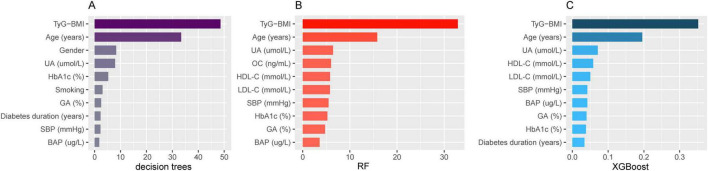
Feature importance with OP in middle-aged and elderly T2DM patients. **(A)** Decision tree; **(B)** random forest; **(C)** extreme gradient boosting. OP, osteoporosis; T2DM, type 2 diabetes mellitus; UA, serum uric acid; HbA1c, glycosylated hemoglobin; GA, glycated albumin; SBP, systolic blood pressure; BAP, bone-specific alkaline phosphatase; OC, osteocalcin; HDL-C, high-density lipoprotein cholesterol; LDL-C, low-density lipoprotein cholesterol.

Three models were constructed based on machine learning algorithms: decision trees, RF, and XGBoost. Using the variables in Table 1 as input variables, the analysis of feature importance distribution revealed that TyG-BMI was the strongest predictor of OP in middle-aged and elderly T2DM patients across all three models (Figure 2).

We used “whether OP occurs or not” as the dependent variable and the variables with a *P* < 0.1 in Table 1 as the independent variables for logistic regression analysis. After adjusting for gender, diabetes duration, smoking, drinking, SBP, HbA1c, GA, TG, HDL-C, LDL-C, P1NP, and BAP, multivariate logistic regression analysis indicated that age, OC, and TyG-BMI were independent risk factors for OP in middle-aged and elderly T2DM patients (OR = 1.147, 95%CI 1.106–1.193, *P* < 0.01; OR = 1.072, 95%CI 1.005–1.149, *P* < 0.05; OR = 1.042, 95%CI 1.031–1.054, *P* < 0.01), while UA served as an independent protective factor (OR = 0.993, 95%CI 0.989–0.997, *P* < 0.01) (Table 2). When stratified by gender, multivariate logistic regression analysis in female group found that age and TyG-BMI were independent risk factors for type 2 diabetic osteoporosis in middle-aged and elderly people (OR = 1.155, 95%CI 1.093–1.231, *P* < 0.01; OR = 1.036, 95%CI 1.020–1.053, *P* < 0.01). Age, HDL-C, and TyG-BMI were independent risk factors for OP in male group (OR = 1.144, 95%CI 1.084–1.217, *P* < 0.01; OR = 7.166 95%CI 1.405–41.843, *P* < 0.05; OR = 1.050, 95%CI 1.033–1.072, *P* < 0.01), while UA was an independent protective factor (OR = 0.989, 95%CI 0.981–0.995, *P* < 0.01) (Table 3).

**TABLE 2:** Multivariate logistic regression analysis for OP in middle-aged and elderly T2DM patients.

Variable	OR	95%CI	*P*
Age	1.147	1.106–1.193	**0.001**
Gender	0.576	0.264–1.236	0.159
Diabetes duration	0.989	0.939–1.042	0.684
Smoking	1.187	0.473–2.990	0.715
Drinking	1.007	0.405–2.476	0.988
SBP	0.996	0.979–1.013	0.641
HbA1c	1.033	0.816–1.309	0.786
GA	1.008	0.950–1.070	0.790
TG	1.056	0.875–1.286	0.576
HDL-C	1.861	0.721–5.120	0.211
LDL-C	1.059	0.786–1.438	0.713
P1NP	1.001	0.985–1.018	0.856
OC	1.072	1.005–1.149	**0.041**
BAP	0.992	0.940–1.046	0.778
UA	0.993	0.989–0.997	**0.001**
TyG-BMI	1.042	1.031–1.054	**0.001**

Boldface indicates statistical significance (*P* < 0.05). SBP, systolic blood pressure; HbA1c, glycosylated hemoglobin; GA, glycated albumin; TG, triglyceride; HDL-C, high-density lipoprotein cholesterol; LDL-C, low-density lipoprotein cholesterol; P1NP, procollagen type 1 N-terminal propeptide; OC, osteocalcin; BAP, bone-specific alkaline phosphatase; UA, serum uric acid; TyG-BMI, triglyceride glucose-body mass index.

**TABLE 3:** Multivariate logistic regression analysis stratified by gender.

Variable	Female			Male		
	**OR**	**95%CI**	***P***	**OR**	**95%CI**	***P***
Age	1.155	1.093–1.231	**0.001**	1.144	1.084–1.217	**0.001**
Diabetes duration	1.011	0.945–1.083	0.758	0.990	0.899–1.085	0.826
Smoking	1.408	0.097–17.114	0.799	1.169	0.408–3.428	0.772
Drinking	1.009	0.071–11.208	0.995	1.439	0.504–4.267	0.500
SBP	0.990	0.967–1.013	0.389	1.004	0.976–1.033	0.789
HbA1c	1.120	0.803–1.594	0.512	0.956	0.659–1.371	0.811
GA	1.000	0.918–1.085	0.994	1.013	0.920–1.114	0.792
TG	1.509	1.041–2.479	0.061	0.889	0.682–1.133	0.350
HDL-C	0.651	0.204–2.467	0.504	7.166	1.405–41.843	**0.022**
LDL-C	0.987	0.654–1.521	0.952	1.081	0.676–1.732	0.743
P1NP	1.010	0.987–1.035	0.394	0.998	0.971–1.023	0.887
OC	1.088	1.000–1.196	0.064	1.038	0.918–1.170	0.546
BAP	1.005	0.929–1.087	0.904	0.971	0.889–1.055	0.488
UA	0.995	0.990–1.000	0.054	0.989	0.981–0.995	**0.001**
TyG-BMI	1.036	1.020–1.053	**0.001**	1.050	1.033–1.072	**0.001**

Boldface indicates statistical significance (*P* < 0.05). SBP, systolic blood pressure; HbA1c, glycosylated hemoglobin; GA, glycated albumin; TG, triglyceride; HDL-C, high-density lipoprotein cholesterol; LDL-C, low-density lipoprotein cholesterol; P1NP, procollagen type 1 N-terminal propeptide; OC, osteocalcin; BAP, bone-specific alkaline phosphatase; UA, serum uric acid; TyG-BMI, triglyceride glucose-body mass index.

**FIGURE 3:**
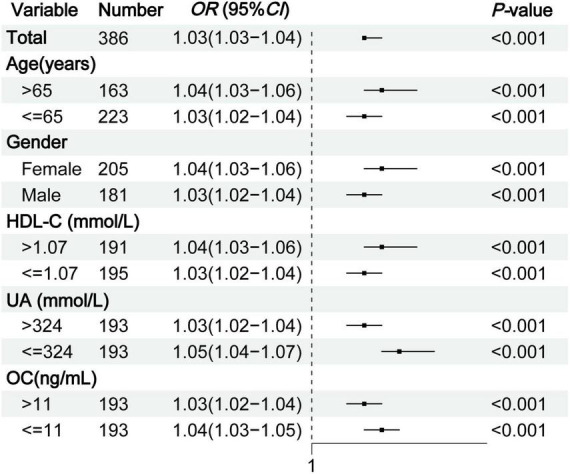
Subgroup analysis of TyG-BMI for OP in middle-aged and elderly T2DM patients. TyG-BMI, triglyceride glucose-body mass index; OP, osteoporosis; T2DM, type 2 diabetes mellitus; HDL-C, high-density lipoprotein cholesterol; UA, serum uric acid; OC, osteocalcin.

Subgroup analysis was conducted according to age, gender, HDL-C, UA, and OC to explore the effect of TyG-BMI on type 2 diabetic osteoporosis in middle-aged and elderly patients. The analysis showed that in all subgroups, the risk of OP increased with higher TyG-BMI levels (all *P* < 0.01), suggesting that TyG-BMI is a stable indicator for predicting OP in middle-aged and elderly patients with T2DM (Figure 3).

### Dose-response relationship between TyG-BMI and the risk of type 2 diabetic osteoporosis

**FIGURE 4:**
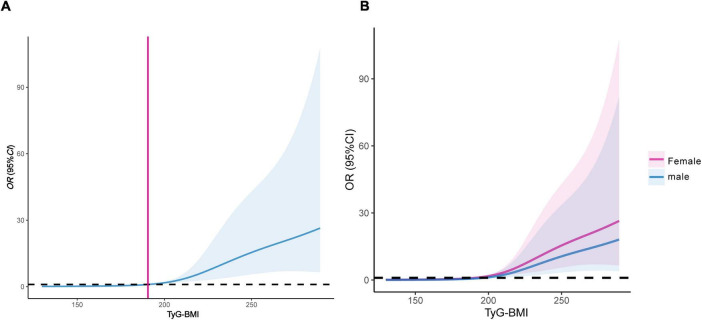
Dose-response relationship between TyG-BMI and the risk of OP in middle-aged and elderly T2DM patients. **(A)** Female and male patients; **(B)** dose-response relationship stratified by gender. TyG-BMI, triglyceride glucose-body mass index; OP, osteoporosis; T2DM, type 2 diabetes mellitus.

After adjusting for multiple confounding factors, RCS showed that TyG-BMI level had a linear relationship with type 2 diabetic osteoporosis in middle-aged and elderly individuals (non-linearity test, *P* > 0.05), and the dose-response relationship revealed an increasing trend. When TyG-BMI > 191.52, the risk of OP in middle-aged and elderly T2DM patients gradually increased (Figure 4A). Stratification by gender showed that when TyG-BMI levels exceeded 186.21 in males and 198.46 in females, the risk of OP gradually increased, with a more pronounced trend observed in female patients (Figure 4B).

### Receiver operating characteristic curves of TyG-BMI for predicting the risk of type 2 diabetic osteoporosis

The clinical utility of TyG-BMI in predicting type 2 diabetic osteoporosis was evaluated using ROC curves. ROC analysis demonstrated a specificity of 84.2% and a sensitivity of 68.5% for identifying the risk of OP in middle-aged and elderly patients with T2DM, with an area under the curve (AUC) of 0.815 (95% CI: 0.771–0.859) (Supplementary Figure 1). Further stratification by gender revealed that in male patients, the specificity was 85.5% and the sensitivity was 66.0%, while in female patients, the specificity was 83.3% and the sensitivity was 74.8%. The corresponding AUC values were 0.781 (95% CI: 0.699–0.864) for males and 0.847 (95% CI: 0.793–0.901) for females (Supplementary Figures 2, 3). These results indicate that TyG-BMI may serve as a valuable predictive marker for assessing the risk of OP in patients with T2DM.

## Discussion

As a bone complication that cannot be ignored in diabetic patients, OP has an insidious onset. In fact, even without typical clinical symptoms, bone strength impairment may already be present. Although BMD is normal or even increased in patients with T2DM, there is an elevated risk of fragility fractures. Once a fracture occurs, it can seriously affect the patient’s quality of life and impose a significant economic burden. Therefore, it is of great clinical significance to explore changes in bone structure in T2DM patients to better prevent and assess the risk of osteoporotic fractures.

The assessment and management of bone health in diabetic patients have been emphasized in the recent ADA guideline (14). A large cohort study published by Taipei University in 2021 on T2DM and OP in Asian populations revealed that the risk of OP was 1.37 times higher in T2DM patients than in the general population (18). This study showed that 42.7% of middle-aged and elderly T2DM patients had OP, with 56.1% of female patients and 27.6% of male patients affected. Compared to a previous study (19), this study found a higher proportion of OP among middle-aged and elderly T2DM patients. A possible explanation is that the subjects in this study were middle-aged and elderly patients, and OP is a degenerative disease associated with aging. As age increases, BMD decreases, and the risk of OP gradually rises. In addition, the subjects in this study were all hospitalized patients who may have experienced long-term hyperglycemia and dyslipidemia, both of which can negatively impact bone health. Studies have shown that blood glucose fluctuations in T2DM patients disrupt the balance between bone resorption and bone formation, leading to changes in bone microstructure and bone loss, thereby increasing the risk of fractures (2, 20). TC, TG, and LDL-C are significantly correlated with OP in postmenopausal women (21), and TC and LDL-C are negatively correlated with heel BMD (22).

A cross-sectional study in 2022 showed that age and UA were independent influencing factors for OP in T2DM patients [OR 95%CI: 1.129 (1.072–1.190), 0.993 (0.988–0.999)] (23). In this study, we demonstrated that age was an independent risk factor for OP in middle-aged and elderly T2DM patients, whereas UA acted as an independent protective factor (both *P* < 0.01). When stratified by gender, UA remained an independent protective factor for OP in male T2DM patients (*P* < 0.01), which was consistent with the findings of Zhao et al. (24). This study also found that HDL-C was an independent risk factor for OP in middle-aged and elderly men with T2DM (*P* < 0.01). Studies showed that increased HDL-C levels in adults in our country were associated with decreased lumbar BMD, particularly in obese men (25), and higher HDL-C levels were associated with an increased risk of fractures in healthy elderly individuals (26).

Previous studies on the correlation between IR and BMD or bone strength in T2DM patients have yielded inconsistent results. Arikan et al. (7), using the HOMA-IR index to assess IR, found that T2DM patients with higher HOMA-IR had lower BMD compared to those with lower HOMA-IR (*P* < 0.05), suggesting a potential negative impact of IR on BMD. A subsequent study in the United States reported that higher HOMA-IR was associated with decreased femoral neck strength but was unrelated to femoral neck BMD (27). However, another study reported a positive correlation between IR metabolic scores and lumbar spine, femoral neck, and hip BMD in postmenopausal T2DM women (all *P* < 0.001) (8). These studies are based on the hypothesis that IR may influence BMD or bone strength in T2DM patients, although some data do not support this hypothesis.

The TyG index is a biological parameter calculated as the product of TG and FPG, and has been proven to be an effective alternative indicator for assessing IR (28). The TyG-BMI is a comprehensive index that combines the TyG index with BMI. In clinical practice, compared to HEC and other screening tests for IR, the TyG-BMI is not only easy to obtain and cost-effective but also provides reliable assessment results. Moreover, it facilitates the early identification and management of individuals at metabolic risk (29). A study conducted in China, which included 832 non-diabetic individuals (474 men aged ≥50 years and 358 postmenopausal women), found a significant association between the TyG-BMI index and OP (adjusted OR: 1.019; 95% CI: 1.01–1.028) after adjusting for age, sex, 25OHD, current smoker, current drinker (30). Notably, this study confirmed that TyG-BMI was an independent risk factor for OP in middle-aged and elderly patients with T2DM. Moreover, a dose-response relationship was observed between TyG-BMI levels and the risk of type 2 diabetic osteoporosis, with cut-off values for TyG-BMI of 186.21 in males and 198.46 in females.

We identified that the predictive value of TyG-BMI for type 2 diabetic osteoporosis stems from its association with IR, glucose toxicity, and lipotoxicity. Firstly, IR is closely related to bone metabolism. Both osteoblasts and osteoclasts contain receptors for insulin and insulin-like growth factor-1. If these receptors are defective, bone-specific IR will result in a significant decrease in the number of osteoblasts and osteogenic function (31), as well as in the expression of the active form of OC, thereby activating osteoclasts and promoting bone resorption (32). Additionally, as IR increases, pro-inflammatory cytokines also increase, which have a greater adverse effect on bone than the anabolic effect of insulin, ultimately leading to a decrease in BMD (33). Secondly, hyperglycemia has negative effects on both osteoblasts and osteoclasts. Hyperglycemia and increased levels of oxidative stress not only elevate the level of advanced glycation end products in diabetic patients, which impacts on bone fragility (34), but also affect the differentiation of mesenchymal stem cells, with fat formation mediated by reactive oxygen species being favored over bone formation (35). Furthermore, lipotoxicity may also negatively affect bone metabolism. Previous studies have found that high levels of free saturated fatty acids enhance the expression of Smurf1, leading to the ubiquitination and degradation of insulin receptors, thereby inducing IR in osteoblasts (36). Excessive fat accumulation in the bone marrow cavity releases free fatty acids, which generate reactive oxygen species that inhibit osteoblast proliferation and function and induce osteoblast apoptosis (37). Lastly, the relationship between obesity and bone health is complex. Maintaining a BMI in the mildly overweight range may optimize bone density (38). Recent findings suggest that being underweight (BMI < 18.5 kg/m^2^) protects Chinese women against spinal fractures, while obesity (BMI ≥ 28 kg/m^2^) increases fracture risk in the spine, lumbar vertebrae, and femoral neck (39). Thus, these clinical and basic research findings explain the potential of TyG-BMI to predict the risk of type 2 diabetic osteoporosis.

The principal advantage of this study is that it is the first to assess the risk of OP in middle-aged and elderly patients with T2DM using TyG-BMI, thereby opening a new direction for studying the correlation between T2DM and OP. We found that a previous study has emphasized the importance of population segmentation in T2DM management (40). In line with this approach, future research could leverage TyG-BMI to stratify T2DM patients into distinct risk categories for OP, thereby enhancing personalized risk assessment and intervention strategies.

However, our study has some limitations. First, this is a single-center retrospective study. Future large-scale, multi-center trials are needed to establish the causal relationship between TyG-BMI and type 2 diabetic osteoporosis. Second, various confounding factors that could influence our findings are not exhaustively considered, such as dietary habits, exercise habits, and sunlight exposure duration. Third, QCT is not a routine clinical tool for assessing OP. In addition, strict exclusion criteria may limit the generalizability of our findings, particularly for patients with comorbidities or those on bone-related medications. Future studies should consider broader inclusion criteria to validate and extend these findings. Finally, machine learning identified TyG-BMI as the strongest predictor of type 2 diabetic osteoporosis. A recent study found that RF had the best predictive performance for diabetes complications (41). We aim to apply machine learning to explore the risk of type 2 diabetic osteoporosis in the future.

## Conclusion

In summary, TyG-BMI demonstrates a positive correlation with type 2 diabetic osteoporosis and serves as a valuable predictor of OP risk in middle-aged and elderly patients with T2DM. This biomarker can be easily calculated using routine laboratory data and anthropometric measurements. These findings highlight the importance of incorporating metabolic health indicators into OP management and warrant further investigation into targeted interventions.

## Data Availability

The datasets presented in this article are not readily available because the data that support the findings of this study were used under license for the current study, and so are not publicly available. Requests to access the datasets should be directed to JZ, zhongjian@hospital.cqmu.edu.cn.

## Supplementary material

Supplementary Figure 1 The ROC curve of TyG-BMI for predicting the risk of type 2 diabetic osteoporosis in middle-aged and elderly patients. ROC, receiver operating characteristic; TyG-BMI, triglyceride glucose-body mass index; OP, osteoporosis; T2DM, type 2 diabetes mellitus.

Supplementary Figure 2 The ROC curve of TyG-BMI for predicting the risk of type 2 diabetic osteoporosis in middle-aged and elderly male patients. ROC, receiver operating characteristic; TyG-BMI, triglyceride glucose-body mass index.

Supplementary Figure 3 The ROC curve of TyG-BMI for predicting the risk of type 2 diabetic osteoporosis in middle-aged and elderly female patients. ROC, receiver operating characteristic; TyG-BMI, triglyceride glucose-body mass index.
