# Supplementary figures and images for: The correlation between TyG-BMI and the risk of osteoporosis in middle-aged and elderly patients with type 2 diabetes mellitus
Source: Front Nutr. 2025 Mar 11;12:1525105. doi: 10.3389/fnut.2025.1525105 (PMC11932904; doi:10.3389/fnut.2025.1525105)

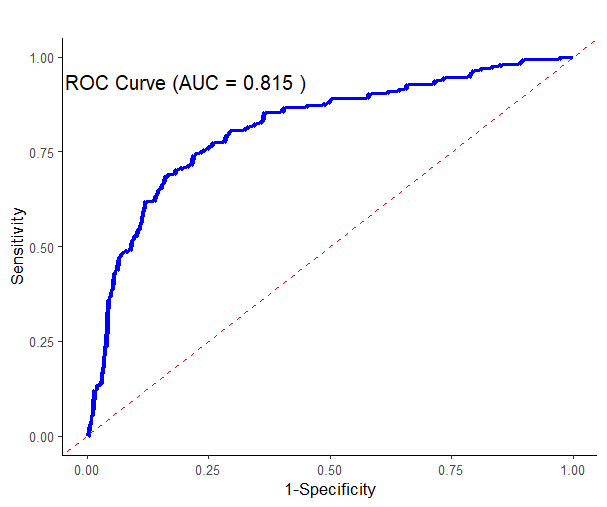

Supplement: Supplementary Figure 1 — The ROC curve of TyG-BMI for predicting the risk of type 2 diabetic osteoporosis in middle-aged and elderly patients. ROC, receiver operating characteristic; TyG-BMI, triglyceride glucose-body mass index; OP, osteoporosis; T2DM, type 2 diabetes mellitus. [file Image_1.tiff]

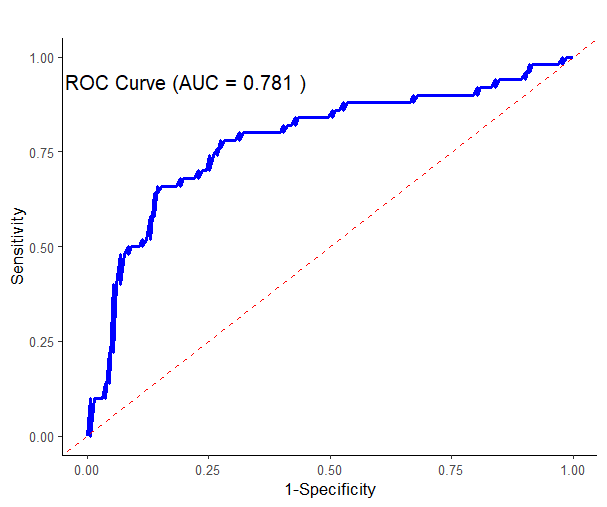

Supplement: Supplementary Figure 2 — The ROC curve of TyG-BMI for predicting the risk of type 2 diabetic osteoporosis in middle-aged and elderly male patients. ROC, receiver operating characteristic; TyG-BMI, triglyceride glucose-body mass index. [file Image_2.tiff]

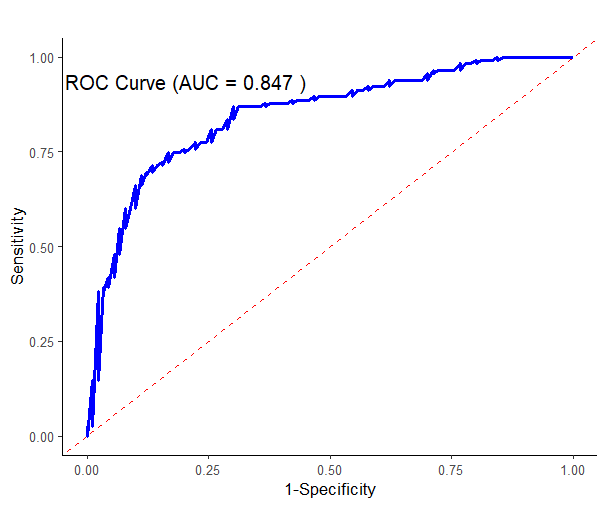

Supplement: Supplementary Figure 3 — The ROC curve of TyG-BMI for predicting the risk of type 2 diabetic osteoporosis in middle-aged and elderly female patients. ROC, receiver operating characteristic; TyG-BMI, triglyceride glucose-body mass index. [file Image_3.tiff]
